# A Model of Photon Cell Killing Based on the Spatio-Temporal Clustering of DNA Damage in Higher Order Chromatin Structures

**DOI:** 10.1371/journal.pone.0083923

**Published:** 2014-01-02

**Authors:** Lisa Herr, Thomas Friedrich, Marco Durante, Michael Scholz

**Affiliations:** 1 GSI Helmholtzzentrum für Schwerionenforschung, Department of Biophysics, Darmstadt, Germany; 2 Technische Universität Darmstadt, Institut für Festkörperphysik, Darmstadt, Germany; Institute of Molecular Genetics IMG-CNR, Italy

## Abstract

We present a new approach to model dose rate effects on cell killing after photon radiation based on the spatio-temporal clustering of DNA double strand breaks (DSBs) within higher order chromatin structures of approximately 1–2 Mbp size, so called giant loops. The main concept of this approach consists of a distinction of two classes of lesions, isolated and clustered DSBs, characterized by the number of double strand breaks induced in a giant loop. We assume a low lethality and fast component of repair for isolated DSBs and a high lethality and slow component of repair for clustered DSBs. With appropriate rates, the temporal transition between the different lesion classes is expressed in terms of five differential equations. These allow formulating the dynamics involved in the competition of damage induction and repair for arbitrary dose rates and fractionation schemes. Final cell survival probabilities are computable with a cell line specific set of three parameters: The lethality for isolated DSBs, the lethality for clustered DSBs and the half-life time of isolated DSBs.

By comparison with larger sets of published experimental data it is demonstrated that the model describes the cell line dependent response to treatments using either continuous irradiation at a constant dose rate or to split dose irradiation well. Furthermore, an analytic investigation of the formulation concerning single fraction treatments with constant dose rates in the limiting cases of extremely high or low dose rates is presented. The approach is consistent with the Linear-Quadratic model extended by the Lea-Catcheside factor up to the second moment in dose. Finally, it is shown that the model correctly predicts empirical findings about the dose rate dependence of incidence probabilities for deterministic radiation effects like pneumonitis and the bone marrow syndrome. These findings further support the general concepts on which the approach is based.

## Introduction

Understanding the dose and dose rate dependence of the cellular response to radiation is of key interest for risk estimations after occupational or accidental radiation exposure as well as for medical applications in radiation oncology. In general, significantly reduced effects are observed after protracted irradiation as compared to acute irradiation where the same total dose is given in a short time of just a few seconds or minutes. Typical dose rates cover a broad range from mGy/year, that are particularly relevant for everyday radiation risk, up to several Gy/min, which are of interest for effects as a consequence of medical applications or severe radiation accidents.

Many studies thus aim to elucidate the impact of specific temporal patterns of dose delivery like e.g. low dose rates, pulsed dose rates or high dose rates [1]–[4] on various endpoints like cell survival probabilities, incident probabilities for diseases and much more [5]–[8].

Induction of DNA damage, in particular double strand breaks (DSBs), has been identified as the key initial event causing observable radiation effects like e.g. cell killing. However, intriguingly the mere number of DSBs is not sufficient to characterize the extent of the effects, since cells in general are able to process and to repair large fractions of the initially induced DNA damage [9]–[13]. It is thus of utmost interest to characterize the type(s) or subset(s) of damage that are less susceptible to repair and consequently lead to an observable effect with higher probability.

Compartmentalization resulting from higher order chromatin structure represents a potential framework allowing for the classification of DSBs with respect to the multiplicity of DSBs in individual substructures. Chromatin loops of 1–2 Mbp genomic length have been identified as relevant for the processing of DSBs [14]–[16]. With regard to the topology of the chromatin loop structure, the induction of multiple DSBs within a loop can be considered as over proportional severe event as compared to induction of a single DSB [17]. Since the probability of inducing severe clustered DSBs will critically depend on the time sequence of induction of individual DSBs and the corresponding repair rates, the mechanistics of dose rate effects are implied by the interplay between DSB induction and repair within chromatin loops and the relevant time scale for dose rate effects is expected to reflect the typical time required for repair of the damage induced. The half-life times representing the typical biphasic exponential decrease of DSBs after an acute irradiation [18]–[20] are considered here to represent the main parameters determining dose rate effects.

The aim of this paper is to present a kinetic model for the assessment of dose response curves based on the approach reported in [17] that analyses the spatio-temporal pattern of DSBs within 1–2 Mbp chromatin substructures. Accounting for the dynamics that result from the interference of damage induction and repair it allows for the calculation of cell survival probabilities after arbitrary irradiation schedules.

In the following, the basic concepts of the model as presented in [17] will shortly be reviewed and the setup of the kinetic extension by introduction of further assumptions will be explained. In order to test the ability to reproduce data – that is, to calibrate the model - fits of the model will be compared to data reported in the literature concerning in vitro dose rate effects [21] and split dose experiments [22]. Furthermore, analytical investigations of the limiting cases of extremely low and high dose rates will be performed and a comparison with the Linear-Quadratic model extended by the Lea-Catcheside factor [23], [24] will be made. Finally, we will demonstrate that the approach is qualitatively predictive with respect to empirical findings concerning clinically observed deterministic effects of radiation. The predictive power of the model after calibration, in terms of an accurate description of cell survival data that has not been used in the fitting routine, has been investigated and will be published elsewhere.

## Models

### Basics of the Giant LOop Binary LEsion model

The Giant LOop Binary LEsion model (GLOBLE) [17] was developed on the basis of the giant loop/random walk model [15], [25], [26] which focuses on the higher order level chromatin organization. Referring to e.g. Yokota et al. [15] and Ostashevsky [25], there are DNA giant loops of about 2 mega base pair (Mbp) size whose terminal ends are attached to the nuclear matrix or fixed in protein complexes. The concept of giant loops was supported by experiments of Johnston et al. [27] who investigated the higher order chromatin structure in a variety of cell lines (humans or rodents, tumorous or normal tissue etc.). In our work, the actual distribution of giant loop sizes amongst and within cell lines will be represented by a constant mean value of 2 Mbp as a first approximation.

The existence of giant loops suggests two categories of radiation induced DNA lesions (binary lesions) with different severity. In case that a single DSB is produced within a loop the two ends of the DNA strand always remain in proximity due to the attachment to their original site. In contrast, if multiple DSBs coexist in a loop, different sizes of DNA fragments might diffuse away and in some instances larger gaps might be opened in the chromatin. Consequently, although the loss of smaller fragments of the DNA might be tolerable for a cell, it should be expected that in the average cellular repair mechanisms run with a much lower fidelity after the induction of multiple DSBs than after the induction of single DSBs. This assumption that the average lethality significantly increases if a second DSB is produced in a loop but only weakly changes with every further DSB, motivates the common classification of multiple DSBs as a “clustered DSB” in contrast to “isolated DSB” in the GLOBLE. Isolated DSBs go in hand with a cell line dependent probability for lethal events (ε_i_) which is much smaller than the probability for lethal events after clustered DSBs (ε_c_). The term “lethal event” comprises a variety of events that lead to cell death, e.g. the inability of a cell to rejoin loose ends or any kind of misrepair of a DSB – potentially involving loop interactions - which has no viable outcome etc.

With ε_i_ and ε_c_ the knowledge about the average number of isolated and clustered DSBs (*n_i_* and *n_c_*) after irradiation is sufficient to calculate the corresponding cell survival probability *S*. It is the Poissonian probability for no lethal event: 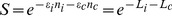
(1)
*L_i_* and *L_c_* denominate the average number of lethal events due to isolated and clustered DSBs, respectively.

In order to assess the numbers of isolated and clustered DSBs after irradiation in the GLOBLE, the DNA giant loops are identified with small target volumes in the cellular nucleus – so called domains [27], [28]. In a first approach there are *N_L_* equally sized domains within a nucleus. Given a genomic length of about 6000 Mbp and an approximate loop size of 2 Mbp one can roughly deduce that *N_L_*≈3000.

As a first working hypothesis with respect to the lethality of DSB classes, domains are supposed to be closed objects. That is, the lethality of damage is fully determined by the number of initial DSBs induced within an individual loop and it does not explicitly depend on the damage induced in the neighboring domains. This does not exclude per se the interaction of DNA ends from different domains and thus e.g. the formation of chromosome aberrations as a consequence of misrepair or misrejoining processes. Lethal events as result of domain interactions are comprised in the mean values ε_i_ and ε_c_.

If instantaneous photon radiation is applied, there is a homogeneous dose deposition over the cellular nucleus. In consistency with our previous studies [17], [29], [30] we assume that DSBs are produced linearly in the dose *D* with a yield of α_DSB_ = 30/Gy/cell. Thus, the average number λ of DSBs per domain is 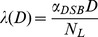
(2)


Exploiting Poissonian statistics to get the probabilities for 0,1,2,… DSBs per domain after the irradiation procedure it holds that: 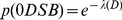
(3)

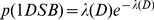
(4)

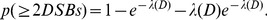
(5)


For the average number of isolated and clustered DSBs it follows that: 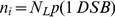
(6)

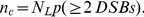
(7)


In [17] the following relation between the lethality coefficients ε_i_ and ε_c_ of the GLOBLE in the origin (*D* = 0) and the coefficients from the Linear-Quadratic model (usually denominated as α and β) has been derived: 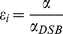
(8)

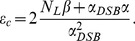
(9)


According to these equations, isolated DSBs are responsible for the initial linear slope of cell survival curves in the GLOBLE and isolated and clustered DSBs affect the quadratic component.

In the derivation of all presented formalisms a constant radiosensitivity over all the phases of the cell cycle has been assumed as a first approach.

### Kinetic extension of the GLOBLE

For the kinetic extension of the GLOBLE, a concept of levels comparable e.g. to laser theory is introduced. In the dynamic process during irradiation, domains transit from one level to another one. To quantify the time dependent occupation of a level, the cell population averaged fraction of domains in a nucleus on the respective level is considered. From the presented premises of the GLOBLE one can derive five levels with corresponding occupations:

*f_0_*: Average fraction of domains with no DSB and no lethal event
*f_i_*: Average fraction of domains with one DSB which has not been processed yet
*f_c_*: Average fraction of domains with more than one DSB which have not been processed yet
*l_i_*: Average fraction of domains with lethal event(s) after processing an isolated DSB
*l_c_*: Average fraction of domains with lethal event(s) after processing a clustered DSB

The distinction of two levels which incorporate domains which have suffered a lethal event is for the sake of comparability with the static formulation of the GLOBLE. Here, the number of domains with a lethal event due to an isolated DSB, *L_i_*, is calculated separately from the number of domains *L_c_* which have suffered a lethal event after a clustered DSB. To be consistent with the working hypothesis that domains are closed objects with respect to the lethality attributed to the different DSB classes, the transition of one domain to another level is independent of the other domains on the same initial level. Since the lethalities comprise lethal events after interaction, there are no quadratic terms but only linear terms involved in the temporal change of the occupation of the levels.

**Figure 1:**
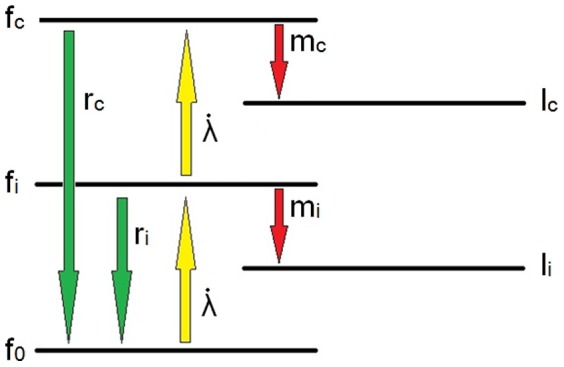
Transitions between levels representing classes of double strand breaks in DNA giant loops. In the GLOBLE there are five levels of DSBs in domains identified with DNA giant loops. These levels are depicted here as solid lines. *f_0_* represents the average fraction of domains without DSB and lethal event. *f_i_* (*f_c_*) represents the average fraction of domains with isolated (clustered) DSBs which have not been processed yet. *l_i_* (*l_c_*) represents the average fraction of domains with lethal event(s) after processing an isolated (clustered) DSB. The transitions between the levels during an irradiation procedure are indicated by arrows. 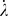 is the rate of DSB induction, *r_i_* and *r_c_* are the rates for repair with viable outcome and *m_i_* and *m_c_* are the rates for the production of lethal events after isolated and clustered DSBs respectively.

To specify the transition pathways involved in the system during and after irradiation the theoretical background of the GLOBLE has to be extended. Figure 1 provides an illustration of the concept. In the kinetically extended GLOBLE one DSB is produced after the other. That is, starting from the level *f_0_* a domain migrates to the *f_i_* level after the induction of a first DSB and afterwards into the *f_c_* level if a next DSB is produced in coexistence with the first one (yellow arrows in figure 1). There is no direct transition from the *f_0_* to the *f_c_* level which however is no severe restriction since the time in which a domain belongs to the *f_i_* level might be infinitely short.

From the two levels where damages have not been processed yet, *f_i_* and *f_c_*, domains migrate back to the *f_0_* level (green arrows) or they migrate to the *l_i_* and *l_c_* level respectively (red arrows) which are “absorbing” (no arrows leave these levels). The former transition means that some repair process has rejoined the DSB(s) within a DNA giant loop with a viable outcome (perfect repair, viable mutation,…). The latter transition means that misrepair has led to at least one lethal event in the corresponding loop and that a viable outcome is no longer possible.

In the kinetically extended GLOBLE, different repair processes with varying repair fidelities are involved in the rejoining of isolated and clustered DSBs respectively. A last DSB remaining after all the other DSBs from a clustered one have been rejoined should not evoke a more effective repair mechanism. Therefore, there is no way to transform a clustered DSB to an isolated one and so there is no transition (arrow) from the *f_c_* to the *f_i_* level.

**Setup of differential equations:** To quantify the time development of the five GLOBLE levels and to finally assess the interference of DSB induction and repair, adequate transition rates have to be found. Motivated by the supposition that DSBs are produced linearly in dose it holds for an arbitrary dose rate 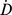 that the average number of DSBs produced within a domain per unit of time is
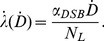
(10)


With this rate domains migrate from *f_0_* to *f_i_* and from *f_i_* to *f_c_* (compare with figure 1).

The processing of DNA DSBs in the nucleus during cellular repair mechanisms should define how the levels *f_i_* and *f_c_* are evacuated again in time. In the kinetic extension of the GLOBLE presented here, we hypothesize that the corresponding transition rates are closely linked to experimentally accessible rejoining rates. Many experiments have revealed evidences for a biphasic rejoining of DSBs where an initial fast component of rejoining (half-life time≈0.5 h) is followed by a much slower one (half-life time≈5 h) [18], [19]. Löbrich et al. found that the slow phase of rejoining is correlated with an increased probability for misrejoining of DSBs compared to the fast phase [31]. Additionally, Johnston et al. hypothesized that the fast component of repair is linked with isolated DSBs whereas the slow component goes in hand with clustered DSBs [14]. We thus identify the half-life time of the fast phase of repair with the half-life time of isolated DSBs *HLT_i_* and the half-life time of the slow phase of repair with the half-life time of clustered DSBs *HLT_c_*. The individual rates *r_i_*, for the transition from the *f_i_* to the *f_0_* level (*f_i_* → *f_0_*), *r_c_* for *f_c_* → *f_0_, m_i_* for *f_i_* → *l_i_* and *m_c_* for *f_c_* → *l_c_* can be gained from the total repair rates (*x* = *i,c*): 
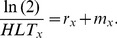
(11)


Due to the fact that the ratio between the lethal misrepair rate *m_x_* and the corresponding total repair rate is the probability for a lethal event (ε_x_) it holds that 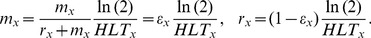
(12)


Putting all the previous statements together yields five coupled differential equations (DEs) in the kinetic extension of the GLOBLE: 
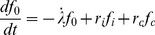
(13)

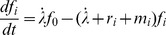
(14)

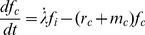
(15)

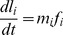
(16)

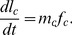
(17)


The survival after an arbitrary irradiation schedule which imposes the adequate initial conditions for the solution of the DEs is defined by 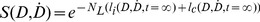
(18)in accordance with equation (1). That is, in the kinetically extended GLOBLE the survival of a cell is determined by the occupation of the levels where domains with a lethal event are accumulated after an infinite amount of time. Since (18) cannot be written in closed form, numerical methods are used in this publication for the application of the model. An approximation allowing for a closed form solution that might be useful in practice is presented in File S1. Furthermore, in File S1 it is shown that the half-life time of clustered DSBs has only little influence on cell survival probabilities and that this parameter can be set to a constant value for calibration purposes or for predictions with the GLOBLE, consequently.

### Single dose treatment with constant dose rate

In case that an irradiation schedule consists in the application of a single dose *D* which is delivered with a constant dose rate 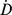 this already defines the protraction time *T*: 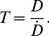
(19)


One can express the occupation of the two levels *l_i_* and *l_c_* after an infinite amount of time as 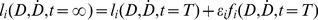
(20)

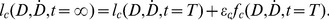
(21)


That is, in order to calculate the average fractions of domains which have suffered at least one lethal event after an infinite amount of time 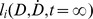 and 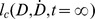 one only has to evaluate the solution of the differential equations (13)–(17) at the point in time *T* where the irradiation ceases. 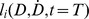 and 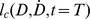 give the average fractions of domains which have already suffered at least one lethal event due to an isolated and a clustered DSB, respectively, up to this point. The average fractions of domains with unprocessed isolated and clustered DSBs at the stopping time (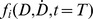 and 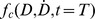 respectively) and the probabilities ε_i_ and ε_c_ define how much more domains will suffer lethal events until infinity. Finally, the occupation of the levels which represent the domains with lethal events after an infinite amount of time is given by the sum of the replenishment during and after the irradiation.

### Split dose experiments

In the split dose experiments considered in this publication cells are treated with two equally sized acute doses *d* separated by a time *t_1_*. If a cellular nucleus contains *N_L_* domains, an acute photon irradiation with dose *d* will produce an average of 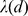 DSBs per domain (equation(2)). Therefore, there is an average of 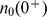, 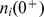 and 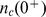 domains without DSB, with an isolated DSB and with a clustered DSB after the first dose has been given at time *t = 0*: 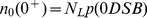
(22)

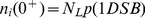
(23)

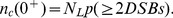
(24)


Here, the upper script “+” denotes “after irradiation” and an upper script “–” will denote “before irradiation” in the following. The probabilities *p(0 DSB)*, *p(1 DSB)* and *p(≥2 DSBs)* can be calculated with equations (3)–(5) (substitute *D* by *d*). Between the two doses there is no induction of lesions but a repair of DSBs takes place. So at time *t_1_* there are
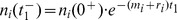
(25)


isolated and
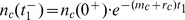
(26)


clustered DSBs left in the average. The expression for the average number of domains without DSB and lethal events 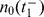 is lengthy and therefore not printed explicitly at this point.

With the probabilities for lethal events after isolated and clustered DSBs, ε_i_ and ε_c_, one can compute the average number of domains that have suffered a lethal event during the repair procedure (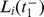 and 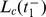): 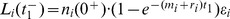
(27)

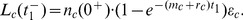
(28)


At time *t_1_*, the second irradiation produces again an average number of 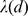 DSBs in each domain in the cellular nucleus. Accounting for the fact that there is already a fraction of domains with isolated or clustered DSBs, the average numbers of domains with isolated DSBs and with clustered DSBs after application of the second dose *d* become: 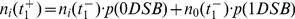
(29)

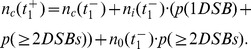
(30)


These DSBs lead to lethal events with probabilities ε_i_ and ε_c_, respectively. Therefore, the total number of lethal events *L* due to a split dose treatment is: 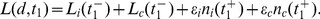
(31)


The corresponding survival probability can be calculated with
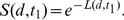
(32)


### Assumptions, experimental data and tools for model performance tests

**Assumptions for the performance tests:** Within the extended framework of the GLOBLE it is assumed that a cell line is characterized by its probabilities for lethal events after isolated and clustered DSBs and by the half-life times corresponding to the two classes of lesions. However, as discussed in File S1, the characteristic features of clustered DSBs imply that their half-life time hardly influences the cell survival probabilities and therefore *HLT_c_* is set to 5 h in the following as a simplification. Moreover, for all the subsequent investigations of the model, the number of domains and the DSB yield are fixed to 3000 and 30/Gy/cell as a first approach. In the end, ε_i_, ε_c_ and *HLT_i_* remain as adjustable parameters. So all cell survival curves recorded for one cell line should be reproducible with one combination of ε_i_, ε_c_ and *HLT_i_* – no matter which dose rate or fractionation scheme was applied during the irradiation.

**Experimental data for the performance tests:** In order to test if the kinetically extended GLOBLE actually describes measured cell survival adequately it was searched for published experiments which demand for the application of kinetic cell survival models for the reproduction of the collected data. Split dose experiments or investigations of the dose rate effect came into consideration. In the end 9 publications which treat 18 cell lines in total were chosen (table 1). Every cell line was irradiated with different dose rates (fourth column) and the corresponding cell survival probabilities were recorded in order to assess dose rate effects. One cell line unexpectedly showed an inverse dose rate effect that is incompatible with the general picture of repair as the relevant factor for dose rate effects and was therefore excluded from the examinations; the authors of the corresponding publication did the same in their own analysis. The other data were used for a first performance test of the GLOBLE. Since the chosen ensemble of investigated cell lines comprises a variety of sources of origin (human, mouse and hamster or normal and tumorous tissue), a good performance of the model in the test should indicate the applicability in the description of cell lines with most diverse characteristics.

**Table 1:** Cell survival data from literature chosen for performance tests.

Publication	Cell line	Expected cell cycle effects	Dose rates [Gy/h]	Doses [Gy]
**Wells and Bedford 1983 ** [**32**]	C3H 10T1/2 (murine embryo)	None^1^	55.6, 2.4; 0.49; 0.29; 0.17; 0.06	
**Stackhouse and Bedford 1993 ** [**33**]	CHO 10B2 (chinese hamster ovary)	None^1^	45; 0.5; 0.12	8+8
**Nagasawa et al. 1989 ** [**34**]	CHO K-1 (chinese hamster ovary)	None^1^	45; 0.153	
**Stisova et al. 2011 ** [**35**]	NFF28 (human fibroblasts)	None^2^	19.98; 0.99	
**Kelland and Steel 1986 ** [**36**]	HX118 (human melanoma)	Unlikely	90; 4.56; 0.96	
	HX32 (human adenocarcinoma of the head and pankreas)	Unlikely	90; 0.96	
	HX99 (human adenocarcinoma of the breast)	Unlikely	90; 4.56; 0.96	
	HX58 (human carcinoma of the pankreas)	Unlikely	90; 0.96	
**Stephens et al. 1987 ** [**37**]	MT (murine mammary carcinoma)	Unlikely	90; 24; 8.4; 4.56; 0.96	5+5; 6+6
	LL (murine lung carcinoma)	Unlikely	90; 8.4; 4.56; 0.96	5+5
	B16 (murine melanoma)	Unlikely	90; 8.4; 4.56; 0.96	5+5
	HX34 (human melanoma)	Unlikely	90; 8.4; 4.56; 0.96	5+5
**Yang et al. 1990 ** [**38**]	IN859 (human glioma)	Unlikely	90; 4.2; 1.2; 0.678	
	IN1265 (human glioma)	Unlikely	90; 4.2; 1.2; 0.678	
	SB (human glioma)	Unlikely	90; 4.2; 1.2; 0.678	
**Ruiz de Almodóvar et al. 1994 ** [**39**]	RT112 (human bladder carcinoma)	Not evident	76.8; 30; 12; 6; 3; 1.2; 0.6	
**Holmes et al. 1990 ** [**40**]	HX138 (human neuroblastoma)	Possible	54; 12; 6; 3; 1.2; 0.6; 0.3; 0.15	
	HX142 (human neuroblastoma)	Possible	54; 12; 1.2; 0.6; 0.3; 0.15	

This table presents experimental data which were chosen for the examination of the kinetically extended GLOBLE. The treated cell lines and the dose rates that were applied during the dose rate effect experiments are listed. In case that survival probabilities after split dose experiments were presented in the publication, the applied doses are noted in the last column. The expected bias in measurements due to cell cycle effects is categorized as follows: “None^1^”: Synchronization in G1; “None^2^”: The population doubling time is much larger than the maximum irradiation time and the applied dose rates make cell cycle effects unlikely; “Unlikely”: The applied dose rates make cell cycle effects unlikely; “Not evident”: The authors state that no bias is evident in the data; “Possible”: The authors state that cell cycle effects cannot be ruled out. For the quantitative investigations of the GLOBLE, the HX99 cell line was excluded. For more explanations see text.

Additionally, 5 of the publications show results of split dose experiments. Here, the specific cell lines were treated with two temporarily separated fractions of equally sized acute doses. Of special interest are the data provided by Stephens et al. [37] and by Stackhouse and Bedford [33] which contain survival probabilities in dependence of the time gap in between the fractions. The applied doses are listed in column 5 in table 1. The corresponding measurements were used for a second performance test of the GLOBLE.

In order to provide information about a possible bias in the measured data due to cell cycle effects, column 3 in table 1 indicates if cell cycle effects should be expected in the considered experiment and if applicable why not. There are three cell lines where cell cycle effects can definitely be ruled out due to synchronization in G1 phase. In one experiment the maximum irradiation time was 3 h which by comparison with the cell population doubling time is unlikely to allow for an observation of cell cycle effects. In reference to Kelland and Steel [36] and Stephens et al. [37] most of the other experiments are also unlikely to be affected by cell cycle effects due to the particular choice of applied dose rates; this will be further described in the discussion of these publications (section “Agreement with experimental data”). Finally, there were only three cell lines where cell cycle effects cannot be ruled out to bias the measured data although for one cell line the authors claime that no bias was observable.

**Tools for the performance tests:** Since all the measured data points (dose | survival probability) or (time of separation | survival probability) are presented graphically in the chosen publications they were read in with “GetData Graph Digitalizer” *(further information to be found at* http://getdata-graph-digitizer.com/ *)*. For stability reasons, the natural logarithm of the survival data and of the computed survival were taken for the execution of fit procedures with the GLOBLE. To find the optimal set of parameters {ε_i_, ε_c_ and *HLT_i_*} for each of the examined cell lines the least squares method was used. For this purpose, the sum of squared residuals over all the data points collected for one cell line was expressed in dependence of ε_i_, ε_c_ and *HLT_i_*. The resulting function was minimized with the “NMinimize” function and the “DifferentialEvolution” method in Wolfram Mathematica 8.0.0 (http://www.wolfram.com/mathematica/).

## Results

In order to validate the concept of the kinetic extension of the GLOBLE, we first assessed the quality of the model in the description of larger sets of experimental data for the two cases of irradiation with constant dose rate and of split dose experiments. We then analyzed the limiting cases of dose rate 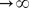 and dose rate → 0 in more detail. Finally, we compared model predictions with empirical formulations of dose rate effects in cell survival curves and in the incidence of deterministic effects.

### Comparison with experimental data

**Figure 2:**
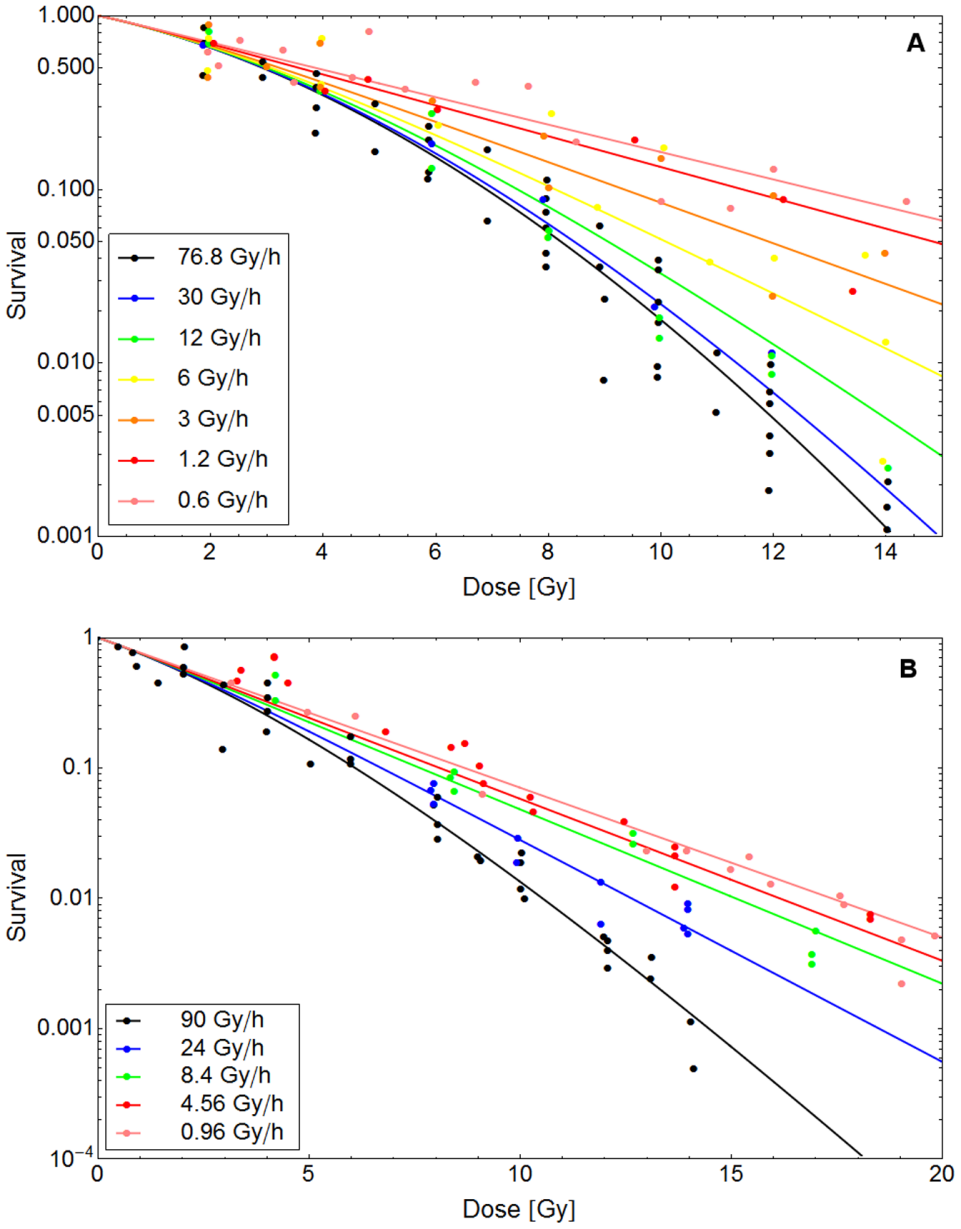
Description of dose rate specific cell survival probabilities with the GLOBLE. The lines in this figure represent fits of the GLOBLE to survival curves of the RT112 (A) and MT (B) cell lines. The experimental data (markers) were taken from [39] and [37].In both experiments the cell lines were treated with different dose rates and the survival probabilities were recorded. In each panel, the measured data are well described by a common set of parameters {ε_i_, ε_c_ and *HLT_i_*} which is – according to the concept – predictive for the cellular response.

**Dose rate experiments:** Figure 2 presents two examples for dose rate experiments. In the data provided by Ruiz et al. and Stephens et al. (dots in the upper and lower panel respectively) [39], [37] it clearly can be seen that the survival probability for a specific cell line after the application of a certain dose increases if the dose rate is lowered. The shape of survival curves is linear-quadratic at high dose rates and becomes more linear with lower dose rates until a completely linear shape is reached.

The measured data in the two panels in figure 2 are described with fits of the kinetic extension of the GLOBLE (lines). In both cases, the experimental results are well reproduced by the model since there are no large deviations between measurement points and the calibrated curves. Obviously, for both experiments it was possible to find an individual set of parameters {ε_i_, ε_c_ and *HLT_i_*} which – according to the concept - allows for the prediction of cell survival probabilities over the whole range of applied dose rates. In other words, for each cell line, there is a characteristic set of parameters {ε_i_, ε_c_ and *HLT_i_*} which determines the effect of any single dose treatment with constant dose rate on this cell line.

In fact, this statement is generally manifested by the other 15 dose rate experiments. In every case a common set of parameters {ε_i_, ε_c_ and *HLT_i_*} describes all the survival curves taken under the application of different dose rates reasonably well. There are no systematic under- or overestimations. The derived parameters ε_i_, ε_c_ and *HLT_i_* reflecting the radiation response of the investigated cell lines are presented in table 2. All the parameter values were found without any constraints in the minimization routine which is in favor of a good applicability of the GLOBLE.

**Table 2:** Parameter values derived from fits of the GLOBLE to experimental data.

	Dose rate experiments			Split dose experiments		
Cell line	ε_i_	ε_c_	HLT_i_ [h]	ε_i_	ε_c_	HLT_i_ [h]
**C3H 10T½**	0.00396	0.0964	2.594			
**CHO 10B2**	0.00130	0.162	6.100	0.00387	0.140	1.337
**CHO K1**	0.00338	0.674	0.0350			
**NFF28**	0.00410	0.455	0.487			
**HX118**	0.0108	0.297	0.236			
**HX32**	0.0142	0.428	5.685			
**HX58**	0.0150	0.425	0.939			
**MT**	0.00865	0.178	0.0859	0.00958	0.119	0.288
**LL**	0.0114	0.543	0.0954	0.0179	0.267	0.458
**B16**	0.00781	0.203	0.131	0.00771	0.180	0.146
**HX34**	0.00893	0.320	0.133	0.0121	0.193	1.095
**IN859**	0.00536	0.407	0.467			
**IN1265**	0.00913	0.215	0.564			
**SB**	0.00490	0.259	0.941			
**RT112**	0.00529	0.195	0.485			
**HX138**	0.0218	0.851	1.184			
**HX142**	0.0284	0.809	1.083			

Here, parameters derived with fits of the GLOBLE to dose rate or split dose experiments in 17 cell lines are listed. These parameter values are – according to the concept – predictive for the radiation response of the investigated cell lines.

As it can be seen in table 2, the values for ε_i_, ε_c_ and *HLT_i_* are reasonable if they are judged by their proposed biological meaning. The two probabilities for lethal events are 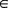
[0;1], ε_i_ is always << ε_c_ and the half-life time of isolated DSBs is smaller than 3 h. The HX32 and the CHO 10B2 cell lines make exceptions featuring half-life times of isolated DSBs which are higher than one would expect from experiments (5.7 h and 6.1 h respectively). However, since peculiar parameter values occur only in these two cases this should not constitute a serious shortcoming. If the two outliers are not accounted for, the median of *HLT_i_* is 0.48 h which is in good agreement with experimental observations.

In the end, there is only one systematic problem in the application of the GLOBLE in dose rate experiments: if cell lines show a dose rate effect despite a linear acute survival curve this can hardly be reproduced or predicted with the kinetically extended GLOBLE. In the model, the linear component of survival curves is dose rate independent and thus linear survival curves with dose rate dependent slopes corresponding to one cell line are not compatible. Optimal fits to the amount of available data sets for such cell lines– in this investigation HX138, HX142 and SB - show too pronounced shoulders at high dose rates to compensate for an initial slope which is too small.

**Figure 3:**
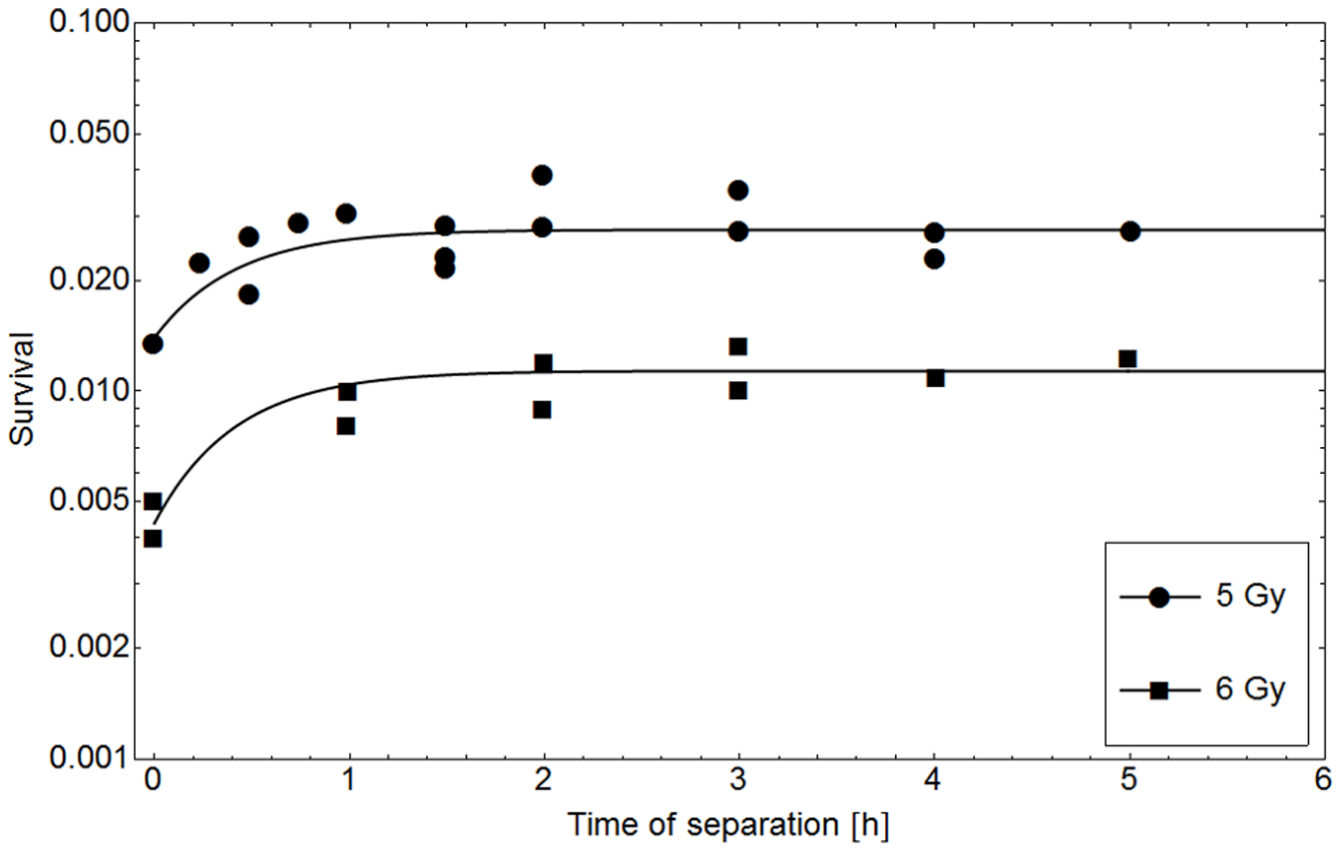
Description of cell survival probabilities after split dose experiments with the GLOBLE. The lines in this figure represent a fit of the GLOBLE to survival curves of the MT cell line. The experimental data (markers) were taken from [37]. In this split dose experiment the cells were irradiated with two times 5 Gy (dots) or two times 6 Gy (squares) and the survival probabilities were recorded in dependence of the time of separation in between the fractions. The measured data are well described by a common set of parameters {ε_i_, ε_c_ and *HLT_i_*} which is – according to the concept – predictive for the cellular response.

**Split dose experiments:** Figure 3 shows the measured survival probabilities (dots and squares) of a MT cell line after it has been irradiated with two equally sized doses (5+5 Gy and 6+6 Gy). With an increasing time between the two fractions the survival probability increases until it finally reaches an upper limit after ∼3 h. Given a certain time of separation the survival probability is higher for lower doses which can be seen in the “level” of the measurement points. The experimental data are described by a single fit of the GLOBLE (lines). Clearly visible, one is able to reproduce the two separation time dependent curves with one common set of parameters {ε_i_, ε_c_ and *HLT_i_*}. There is no systematic deviation between the modeled graphs and the measured points in terms of an under- or overestimation of the survival in certain sections of the curves.

Fits to split dose experiments in four other cell lines verify the good performance of the GLOBLE in terms of a distinct agreement of measured data points with the describing graphs. Furthermore, table 2 shows that the parameter values resulting from the fits are in accordance with what one would expect from their biological meaning. The probabilities for lethal events are 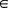 [0;1], ε_i_ is always << ε_c_ and the half-life times for isolated DSBs are in the range of experimental observations with a median of 0.458 h. There are no exceptions or peculiarities which would argue against the applicability of the GLOBLE in split dose experiments.

### Analytical examinations

**Limiting case: Dose rate** 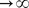 **:** If the applied constant dose rate 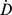 in a single dose treatment becomes extremely large (with fixed protraction time *T*) this means that the dose *D* is given in an instant to the target. Thus, in the limit 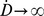 the kinetically extended GLOBLE should converge to the static version of the model published in [17]. Actually, this is the case as it will be shown in the following.

For 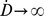 all rates in the differential equations (13)–(17) become negligible in comparison to 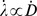 so that (13)–(14) reduce to 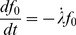
(33)

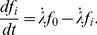
(34)


Exploiting the fact that 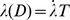 and that due to the conservation of domains *f_c_* = 1−*f_0_* − *f_i_*, the solution of these differential equations and the expression of *f_c_* is the same as equations (3)–(5). Therefore, the kinetic extension of the GLOBLE merges into the already published static version for extremely large dose rates.

**Limiting case: Dose rate → 0:** If a single dose is applied with a very low constant dose rate there is a large amount of time for the processing of a first DSB before the next lesion occurs within the same domain. Since there will be no coexistence of two or more DSBs in one domain, no clustered DSBs will be produced during the irradiation. For the system of differential equations (13)–(17) this means that every contribution linked with a clustered DSB has to be eliminated. This yields 
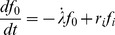
(35)

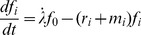
(36)

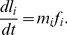
(37)


Given no DSBs at the beginning of irradiation, the survival probability derived from the solution of this system of differential equations is implied by: 
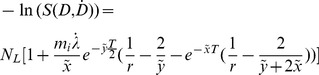
(38)with
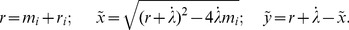
(39)


The first order Taylor expansion coefficient of the solution equals ε_i_α_DSB_ which is consistent with the static case and equation (8). It dominates the shape of survival curves at low dose rates. The very small second order Taylor expansion coefficient at such low dose rates is negative and thus implies a slight upwards bending of cell survival curves. This effect can be explained with the fact that a second lethal event – the corresponding probability increases with the dose - causes no further harm in domains which have already suffered a lethal event. Consequently, survival probabilities decline less and less at higher doses. However, since the linear component of the curves is much larger than the quadratic contribution at low dose rates (in the linear quadratic framework compare e.g. α = 0.15/Gy to β = −10^−6^/Gy^2^), the upwards bending of survival curves predicted with the GLOBLE is of no practical relevance.

### Comparison with empirical findings

**Dose rate effect in cell survival probabilities:** In cell survival studies, treatment planning and many other areas it is common to quote parameters of the Linear-Quadratic model (LQ) for the characterization of a treated cell line. Therefore, comparing the kinetically extended GLOBLE with the LQ and relating the newly developed parameters with the established ones is instructive.

The introduction of the Linear-Quadratic model (LQ) for the description of cell survival after instantaneous irradiation was mainly motivated by the empirical observation that cell survival curves typically show a linear and a quadratic component if they are plotted on a logarithmic scale. Consequently, in the LQ, the negative natural logarithm of the survival is a polynomial of second order in the dose: 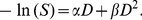
(40)


It can be adjusted for a reproduction of dose rate effects in cell survival probabilities occurring when a single dose *D* is stretched over a protraction time *T* [23], [24]: 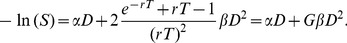
(41)


In the following, we will always refer to this adjusted version if we consider the LQ. The rate *r* represents the linear restitution of chromosome aberrations in time in the original publication. Therefore, in a more general sense, only the kind of lesions which is responsible for the linear slope of the LQ survival curves enters the Lea-Catcheside factor *G*.

**Figure 4:**
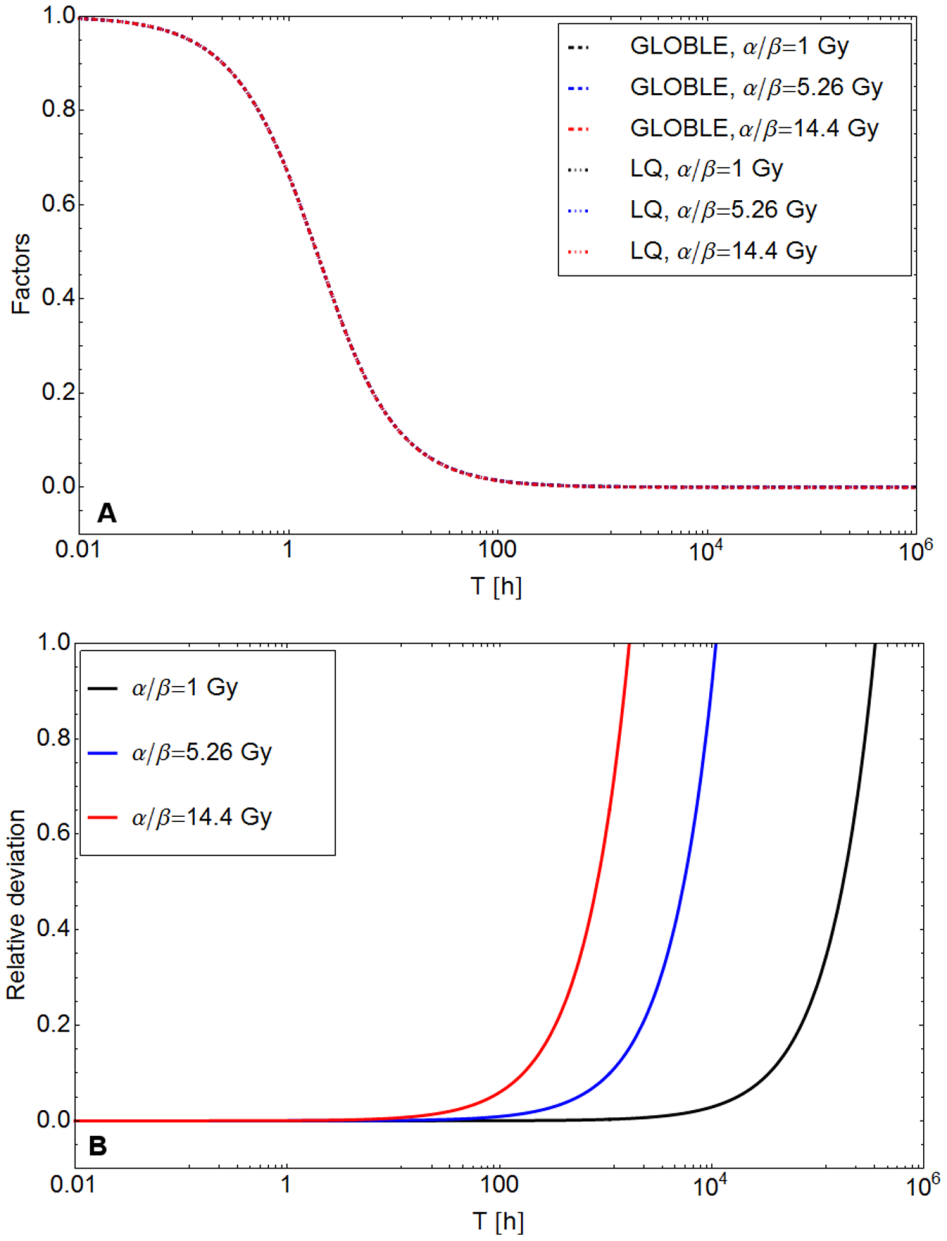
Comparison of the GLOBLE and the Linear Quadratic model extended by Lea and Catcheside. A: The Lea-Catcheside factor and its GLOBLE-equivalent are plotted in dependence of the protraction time *T*. Three hypothetical cell lines with different values of α/β were chosen. The employed parameters are: α/β = 1 Gy with α = 0.025/Gy, β = 0.025/Gy^2^, ε_i_ = 0.00083, ε_c_ = 0.17; α/β = 5.26 Gy with α = 0.15/Gy, β = 0.0285/Gy^2^, ε_i_ = 0.002, ε_c_ = 0.2; α/β = 14.4 Gy with α = 0.36/Gy, β = 0.025/Gy^2^, ε_i_ = 0.012, ε_c_ = 0.19. For all α/β: *r*  =  *(r_i_ + m_i_)*  =  ln(2)/(0.5 h) and *(r_c_ + m_c_)*  =  ln(2)/(5 h). Please note that all the lines lie on top of each other. B: The relative deviation of the Lea-Catcheside factor and its GLOBLE-equivalent are plotted over the protraction time *T* for different values of α/β.

Through the introduction of the Lea-Catcheside factor the quadratic contribution to modeled cell survival curves diminishes with an increase in the protraction time *T*. This implies an increase in cell survival probabilities until maximum values are reached with straight survival curves. Figure 4 shows the decrease of *G* (dotted lines) from 1 at small to 0 at large protraction times. *G* = 1 implies a full quadratic bending of survival curves after acute irradiation (small protraction times) and *G* = 0 implies straight survival curves.

For a comparison with the LQ model, the dose rate 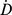 in the negative logarithm of the survival in the GLOBLE formalism was substituted by *T* with equation (19). The coefficients of a Taylor expansion in the origin (*D* = 0) up to second order which are equivalent to α (first order coefficient) and 2*G*β (second order coefficient) were derived. Independent of the protraction time, the first order coefficient equals the one that has already been found in the examination of the static GLOBLE. That is, no matter which protraction time is chosen for an irradiation, the initial linear slope of cell survival curves remains constant. This is comparable to the LQ and the identification of the first order coefficient at *D* = 0 with α yields α  =  ε_i_α_DSB_ in agreement with equation (8).

The second order Taylor expansion coefficient of *−ln(S)* at *D* = 0 varies with *T* in the kinetically extended GLOBLE. Since its mathematical formulation is longish it is not presented explicitly at this point. It is dominant in the prediction of the dose rate effect – i.e. the disappearance of shoulders in cell survival curves if one increases the protraction time of the irradiation. To find an equivalent for the Lea-Catcheside factor *G* the calculated second order coefficient of the GLOBLE was multiplied with 0.5 and normalized with the GLOBLE expression for β implied by equations (8)–(9). Since the rate *r* in the kinetic extension of the Linear-Quadratic model incarnates only the lesions which are responsible for the linear component of cell survival curves this rate *r* was identified with *(r_i_ + m_i_)* – the total rate of repair of isolated DSBs which produce the initial linear slope in the GLOBLE.

Figure 4 illustrates that the Lea-Catcheside factor and its GLOBLE-equivalent are almost equal in a wide range of protraction times. The two factors were calculated for hypothetical cell lines with three different α-β combinations. Equations (8)–(9) were used to convert α and β into ε_i_ and ε_c_. In reference to experimental observations and values derived with the model above, the value for *r  =  (r_i_ + m_i_)* was set to ln(2)/(0.5 h) and *(r_c_ + m_c_)* was chosen to be ln(2)/(5 h). The upper panel (A) makes clear that the differences between the Lea-Catcheside factor and its GLOBLE-equivalent are hardly noticeable by eye. Therefore the lower panel (B) additionally gives the relative deviation of the two factors. It starts to increase rapidly to infinity at large protraction times – sooner for high α/β ratios and later for low α/β ratios. This is because the GLOBLE features a finite second moment at low dose rates (equivalent to long protraction times) whereas the LQ second moment approaches 0. However, it has to be emphasized that the large relative errors are of no practical relevance as they affect only the values which are anyway almost identical to zero.

Although the GLOBLE and the Linear-Quadratic model are almost equal up to second order there are distinct differences in the reproduction or prediction of cell survival probabilities as it was already stated in [17]. The GLOBLE features a saturation effect at higher doses whereas cell survival probabilities decline ever more in the LQ model due to the dominating quadratic component. It was found that the cause for larger deviations between the GLOBLE and the LQ model are due to contributions of higher orders in dose.

**Incidence of deterministic effects of radiation:** Cell killing is considered to be a major determinant of deterministic effects that are observed in tissues after radiation insult [41], [42]. Therefore, the approach presented in this publication was expected to be applicable also to describe dose rate effects for these endpoints. Although a detailed comparison to the relevant clinical data was beyond the scope of this paper, we roughly assessed the order of magnitude of dose rate effects predicted with the GLOBLE and with an empirical relationship that is recommended for use in the field of radiation protection.

Empirically, it has been observed that the dose *D_50_* at which 50% of the exposed persons show defined clinical symptoms of deterministic effects linearly increases with a decrease of the dose rate. This can be expressed in terms of a linear function of the inverse of the dose rate [43]: 
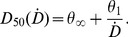
(42)


**Figure 5:**
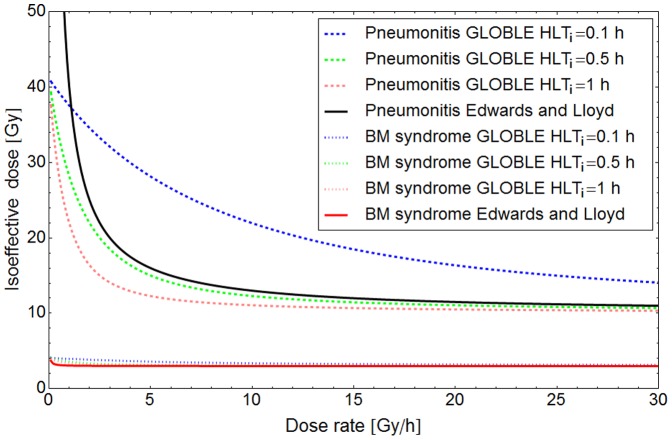
Prediction of the dose rate dependence of deterministic radiation effects. The solid lines show the empirical dose rate dependence of the dose implying a 50% probability for the incidence of pneumonitis and the bone marrow syndrome after an exposure as presented by Edward and Lloyds [43]. The order of magnitude of the dose rate dependence can be predicted with the GLOBLE if a reasonable range of half-life times of isolated DSBs *HLT_i_* from 0.1 h to 1 h is assumed (dashed and dotted lines). The shape of the empirical curve for pneumonitis is in good agreement with the GLOBLE prediction down to ≈3 Gy/h if *HLT_i_* is set to 0.5 h which corresponds to the median of half-life times derived from in vitro data.

The parameters 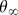 which equals *D_50_* after an acute irradiation and 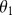 which reflects the extent of the dose rate effect are characteristic for a considered disease. Exemplarily, figure 5 shows the dose rate dependence of *D_50_* for two extreme cases, namely pneumonitis and the bone marrow syndrome (black and red solid line). For pneumonitis values of 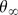  =  10 Gy, 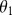 = 30 Gy^2^/h are reported, whereas the bone marrow syndrome is represented by 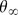 = 3 Gy, 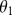 = 0.07 Gy^2^/h. Accordingly, one sees a large increase of *D_50_* for pneumonitis if the dose rate is lowered but only a small shift of *D_50_* in case of the bone marrow syndrome.

For a comparison with the GLOBLE, ε_i_ and ε_c_ were derived by means of equations (8) and (9) from the linear-quadratic parameters corresponding to the endpoint under consideration. For pneumonitis, an α/β ratio of 3 Gy is reported [44], whereas for the acute bone marrow syndrome an α/β ratio of 8 Gy is found [6]. It is important to note here that the dose rate effect in our model only depends on the ratio ε_i_/ε_c_ and with that via equations (8) and (9) on the α/β ratio, but not on the absolute parameter values. The values used for the calibration of the GLOBLE were ε_i_ = 0.00333 and ε_c_ = 0.229 for pneumonitis and ε_i_ = 0.00333 and ε_c_ = 0.09 for the bone marrow syndrome. To find an endpoint equivalent to *D_50_* with the GLOBLE, the dose leading to the same cell survival probability as the instantaneous application of 10 Gy (for pneumonitis) and 3 Gy (for the bone marrow syndrome) was taken (isoeffective dose).

In figure 5 it can be seen that the order of magnitude of the dose rate effect is the same for the empirical formula (42) (solid lines) and for predictions with the GLOBLE (dotted lines). A significant dose rate dependence is predicted in the case of pneumonitis but only a very weak dependence in the case of the bone marrow syndrome. With a half-life time of isolated DSBs of 0.5 h motivated by experiments and the median values derived above (green dotted and dashed lines) one even finds an agreement of the shape of equation (42) and the GLOBLE down to dose rates of about 3 Gy/h for pneumonitis. A disagreement at lower dose rates has to be expected since *D_50_* in equation (42) diverges for infinitely low dose rates whereas the isoeffective dose converges to a finite value implied by straight cell survival curves in the GLOBLE.

## Discussion

In the previous sections it has been shown that the kinetically extended GLOBLE is an appropriate model for the assessment of cell survival after photon irradiation with arbitrary dose rates. In the following we will briefly discuss the results amongst others by pointing out conceptual differences to other models and by comparing the repair rates found in the results section to other published data.

### The concept of the GLOBLE in comparison to other cell survival models

The GLOBLE differs from other approaches in the modeling of cell survival due to a strong commitment to empirical findings. Not only the input parameters but as well the introduced classes of lesions are strongly linked to biological mechanisms which were deduced from experimental observations. In particular the introduction of two discrete lesion classes namely isolated and clustered DSBs that are characterized on the basis of the multiplicity of DSBs in higher order chromatin structure represents a distinct feature of the model. In the GLOBLE cell survival thus is not directly expressed in dependence of the dose, as it is common in the setup of many other cell survival models. Instead, it is primarily the spatio-temporal damage distribution pattern that is used to determine the cell killing probability. At first, the distinction between dose deposition and damage distribution pattern might appear to be subtle. However, actual differences in the dose deposition pattern do not necessarily lead to differences in the damage distribution pattern. That is, completely different local and temporal dose depositions might produce the same amount of isolated and clustered DSBs and therefore evoke the same radiation effect.

In this regard, the kinetic approach proposed in this publication suggests an extension to other radiation qualities. Whereas we have been focusing here on applications to photon radiation, an extension to high-LET radiation is straightforward and will thus be an essential part of future investigations. Since parts of the presented concepts are also implemented in the Local Effect Model (LEM) [29], [30] which allows to predict the increased effectiveness of acute high-LET irradiations, the LEM is considered to be particularly suitable for such an extension. Since model parameters should reflect cell specific (repair) characteristics, the potential global use of one set of parameters for all radiation qualities might constitute a major advantage of the concept of the classification of DSBs into isolated and clustered ones.

As already indicated, there might be some clustered DSBs composed of fragments which are too small to allow for a distinction from isolated DSBs in terms of their individual lethality. However, in the special case of photon radiation which has been considered in this publication there is a homogenous dose deposition over the cellular nucleus which implies an almost homogenous damage distribution. Therefore, the yield of extremely small fragments should be negligible in a first approximation. With regard to modeling cell survival after ion radiation the introduction of a threshold for fragment sizes defining a clustered DSB might be necessary due to the high density of lesions along the particle tracks.

A detailed qualitative and quantitative comparison of the kinetically extended GLOBLE with other already established kinetic cell survival models e.g. the Incomplete Repair model (IR) by Oliver et al. [45] or the Lethal Potentially Lethal model (LPL model) by Curtis [46] might be of interest but is beyond the scope of this publication. Nevertheless, already at this stage, one might consider reported results from fits of the IR model or the LPL model to experimental data for a rough assessment of the degree of accordance with fit results from the GLOBLE. In some of the publications chosen for the performance tests of the GLOBLE, the IR model or the LPL model were used for fits to dose rate experiments. For these cases estimated half-life times for lesions defined in the IR model and the LPL model which might be compared to *HLT_i_* from the GLOBLE are directly at hand (column 3, 5 and 6 in table 3). Obviously, the half-life times found with the IR model and the LPL model in dose rate experiments are in the same order of magnitude as the ones determined with the GLOBLE.

**Table 3:** Comparison of half-life times of DSB repair determined with different models.

		GLOBLE			
Cell line	Experiment	Dose rate exp.	Split dose exp.	IR	LPL
**CHO 10B2**	1.17	6.10 (+421.4%)	1.34 (+14.5%)		
**HX118**	0.42	0.24 (−42.9%)		0.23 (−45.2%)	0.32 (−23.8%)
**HX32**	2.02	5.69 (+181.2%)		5.01 (+148.0%)	3.57 (+76.7%)
**HX58**	1.42	0.94 (−33.8%)		0.8 (−43.7%)	0.68 (−52.1%)
**MT**	0.19	0.09 (−52.6%)	0.29 (+52.6%)	0.094 (−50.5%)	0.104 (−45.3%)
**LL**	0.61	0.10 (−83.6%)	0.46 (−24.6%)	0.092 (−84.9%)	0.069 (−88.7%)
**B16**	0.16	0.13 (−18.8%)	0.15 (−6.3%)	0.13 (−18.8%)	0.123 (−23.1%)
**HX34**	0.97	0.13 (−86.6%)	1.10 (+12.4%)	0.117 (−87.9%)	0.105 (−89.2%)
**RT112**	0.93	0.48 (−48.4%)			
**HX138**	1.0	1.18 (+18.0%)		1.13 (+13.0%)	
**HX142**	1.6	1.08 (−32.5%)		1.22 (−23.8%)	

Different cell line specific half-life times of DSB repair in h are presented in this table. The experimental values were gained with exponential recovery fits to split dose experiments [21], [33], [36], [37]. The listed values resulting from a fit of the Incomplete Repair model (IR model) or the Lethal Potentially Lethal model (LPL model) to cell survival curves are taken from the following publications: [36], [37], [40]. The values given for the GLOBLE (which correspond to *HLT_i_*) were determined with fits to experimentally measured cell survival data as shown in the results of this paper. The relative deviation of the half-life times derived with the models to the experimental one are written in parentheses.

### Cluster effects on micrometer versus nanometer scale

In the GLOBLE approach presented in this publication the clustering of DSBs within DNA giant loops is considered to be decisive for cell survival probabilities. That is, the existence of multiple DSBs within chromatin structures of about 2 Mbp or within a few micrometers is supposed to drastically increase the risk for cell death compared to single lesions within such orders of magnitude. This hypothesis is strengthened by findings of Johnston et al. who state that chromatin loops in the order Mbp are the “critical targets for induction of DSBs” [27], and that the damage distribution in those units might influence the radiation response of a cell. Furthermore, the authors found that multiple lesions within the looped structures are processed with slow kinetics and that they are “more resistant to repair than individual DSBs” [47]. The results of Gauter et al. [48] confirm that after a random initial distribution of DNA fragments it is fragments of < 2–3 Mbp size that dominate the slow component of repair and that this fraction of slowly repaired DSBs increases with the applied dose. The analysis presented by Tommasino et al. further supports the view of fast processing of iDSB and slow processing of cDSB for both low- and high-LET radiation [49]. Together with the observation of Löbrich et al. [31] that the slow component of repair is correlated with an increase in misrejoining the argumentation for the decisiveness of DNA giant loops for cell killing is strengthened.

On the other hand, there are indications that the clustering of DNA damage including single base damages, strand breaks and double strand breaks within a few nanometers is important for the effectiveness of radiation. Nikjoo et al. [50] simulated that the complexity of clustered damage on nm scale increases with the LET of the radiation and that it is locally confined to a few base pairs. In agreement with these findings are calculations by Ottolenghi et al. [51] showing that the characteristics of ion radiation damage is crucially dependent on the track structure and that a categorization of DSBs in terms of complexity on nanometer scale allows to find a correlation between DSBs involving deletions with the RBE. Therefore, the properties of DSBs on nanometer scale should be considered if the effectiveness of radiation is determined. Finally, the impact of locally clustered damage in connection with the implied challenge for cellular repair mechanisms is pointed out by Ward [52]. He concludes that the distinct shape of local DNA damage is important for an assessment of its lethality due to the cell line specific repair processes on this level.

Modeling work directly comparing short scale and long scale clustering effects as reported by Friedland and co-workers [53] showed that a better correlation to the experimental cell inactivation data was found for DNA damage clustering at a regional scale compared to the local scale. It can thus not be excluded that, depending on the particular assay, endpoint and radiation quality, clustering on both levels might be relevant to characterize the biological effectiveness of a specific radiation quality.

Although damage clustering on the nm scale is not explicitly accounted for in the GLOBLE, this should not reject the possibility for its influence on cell survival. Since in the GLOBLE we refer to measured DSB yields, these implicitly include the whole spectrum of complexities (on the nm scale) that can be attributed to individual DSBs, and the fact that only a certain fraction of DSBs has a high impact on cell killing might be reflected in the value of ε_i_ << 1, which thus has to be regarded as an effective lethality for all (clustered on the nm-scale and non-clustered) isolated DSBs. However, for photon radiation the fraction potentially attributable to clustered lesions on the nm scale is unlikely to change within the experimental relevant range of doses and dose rates, since presumably all DSBs are induced by single track effects as indicated by the linear dependence of the yield on dose. When the concept of the GLOBLE will be used to model ion radiation effects, the lesion induction concept of the LEM may be used. There an enhanced DSB yield compared to photons is resulting from an explicit simulation of single strand break clustering on the nm scale. In conclusion, clustering of damage on the nanometer versus micrometer scale is not contradictory in the concept of the GLOBLE but rather complementary.

### Agreement with experimental data

As demonstrated in the results section, the GLOBLE allows for a reproduction of low LET cell survival probabilities after application of protracted or split doses. Since there are no systematic deviations, the simplifications made as a first approach seem to be reasonable. For instance, the assumption of a constant, average radiosensitivity over all phases of the cell cycle does not strongly bias the final results although in principle cell cycle dependent radiosensitivities have to be expected. We found that there are no significant differences in the quality of the description of the chosen experiments where cell cycle effects can be excluded with varying confidence or cannot be excluded at all.

One reason why distortions due to cell cycle effects should not be expected in in most of the considered experiments anyway is given by the relatively long population doubling time of the investigated cell lines in comparison to the maximum irradiation time. This statement is amongst others supported by Kelland and Steel [36] and by Stephens et al. [37]. Another reason is again linked to the choice of dose rates. At high dose rates, cell cycle effects in terms of an increase in radioresistance after the depletion of cells being in radiosensitive phases cannot be measured. At low dose rates there is only a small effectiveness in cell killing and thus the heterogeneity of a cell population and its average radiosensitivity might be maintained over the long irradiation time. Finally, only intermediate dose rates might allow for some bias in measurements due to cell cycle effects.

A next point that might be discussed is the assumption of a constant DSB yield of 30/Gy per cell. In experiments the range of measured DSB yields goes from about 25/Gy per cell [54] up to about 60/Gy per cell [55]. Our results suggest that the employment of an intermediate value as it is given by 30/Gy per cell is a reasonable approach. Potential variations in the DSB yield amongst different cell lines or amongst different stages of the cell cycle do not lead to systematic distortions. The same holds true for variations in the genomic length of different cell lines. The implied adjustment of the number of domains per cell would be in the order of up to 10% which does not lead to a significant variation in the model output, since variations e.g. in the number of domains can be compensated by a corresponding variation of the lethalities ε_i_ and ε_c_; the same holds true for a variation of the DSB yield.

### Comparison of reported and derived half-life times of the fast component of repair

In order to further assess the consistency of the mechanistic ideas behind the GLOBLE approach, we will compare the corresponding half-life times with other observations reported in the literature.

For instance, the half-life times for isolated DSBs (*HLT_i_*) can be compared to published half-life times of the fast component of repair. Table 3 shows a list of cell lines whose half-life times of repair were determined in split-dose experiments and calculated with exponential recovery fits (column 2) [21], [33], [36], [37]. These values are compared to the results from the GLOBLE fits to dose rate and split dose experiments with the same cell line. As it can be seen, the experimental results for the cell line dependent half-life times of repair (exponential recovery) are roughly in the same order of magnitude as the GLOBLE half-life times for isolated DSBs. Only the fits to dose rate experiments with HX32 and the CHO 10B2 cell lines produce larger deviations (181.2% and 421.4% respectively) as it was already expected after the investigation of the results.

Furthermore, table 3 reveals that half-life times of isolated DSBs resulting from GLOBLE fits to dose rate experiments (column 3) generally underestimate the half-life times gained with an exponential recovery fit (with exception of the two outliers and one other cell line). If *HLT_i_* is determined with GLOBLE fits to split dose experiments (column 4), these values are much closer to the values gained by an exponential recovery fit to the same experimental data – as it should be expected. There is no systematic under- or overestimation.

Obviously, the half-life times found with the IR model and the LPL model in dose rate experiments (column 5 and 6) underestimate the values found in split dose experiments in the same order of magnitude as the ones determined with the GLOBLE. There seems to be a common disagreement of theory and measurements which amongst others Stephens et al. and Steel et al. already pointed out in their publications of 1987 [37], [21]. Stephens et al. propose that the reason for the discrepancy might be the inability to maintain a constant temperature during split dose experiments which is an important condition for unbiased results. Steel et al. suggest that the fast component of repair might not be assessable in split dose experiments whereas it dominates in dose rate experiments. Therefore, an exponential recovery with a single half-life time which “averages” over potentially existent multiple components of repair leads to other results than models accounting for more than one component of repair.

During our investigation of the GLOBLE, we came to similar conclusions as Steel et al. Deviations of the GLOBLE parameter *HLT_i_* from the half-life times gained with an exponential recovery fit might be reasoned with the different approaches in taking into account cellular repair processes. In the GLOBLE there are two classes of lesions which are repaired with different fidelities and kinetics whereas in the exponential recovery there is only one kind of lesion with a purely exponential decrease in the time after irradiation. Although the magnitude of the half-life time of clustered DSBs *HLT_c_* is of little importance (as explained in more detail in File S1), the mere existence of a second component of repair is highly relevant. With increasing dose and dose rate, the balance between isolated and clustered DSBs is shifted towards clustered DSBs and the fraction of isolated DSBs susceptible to fast repair consequently decreases. Due to the different amount of initial damage which is finally linked to the considered component of repair – in case of the GLOBLE the fast one - large deviations between single and double exponential repair might be expected, depending on the irradiation scheme.

**Figure 6:**
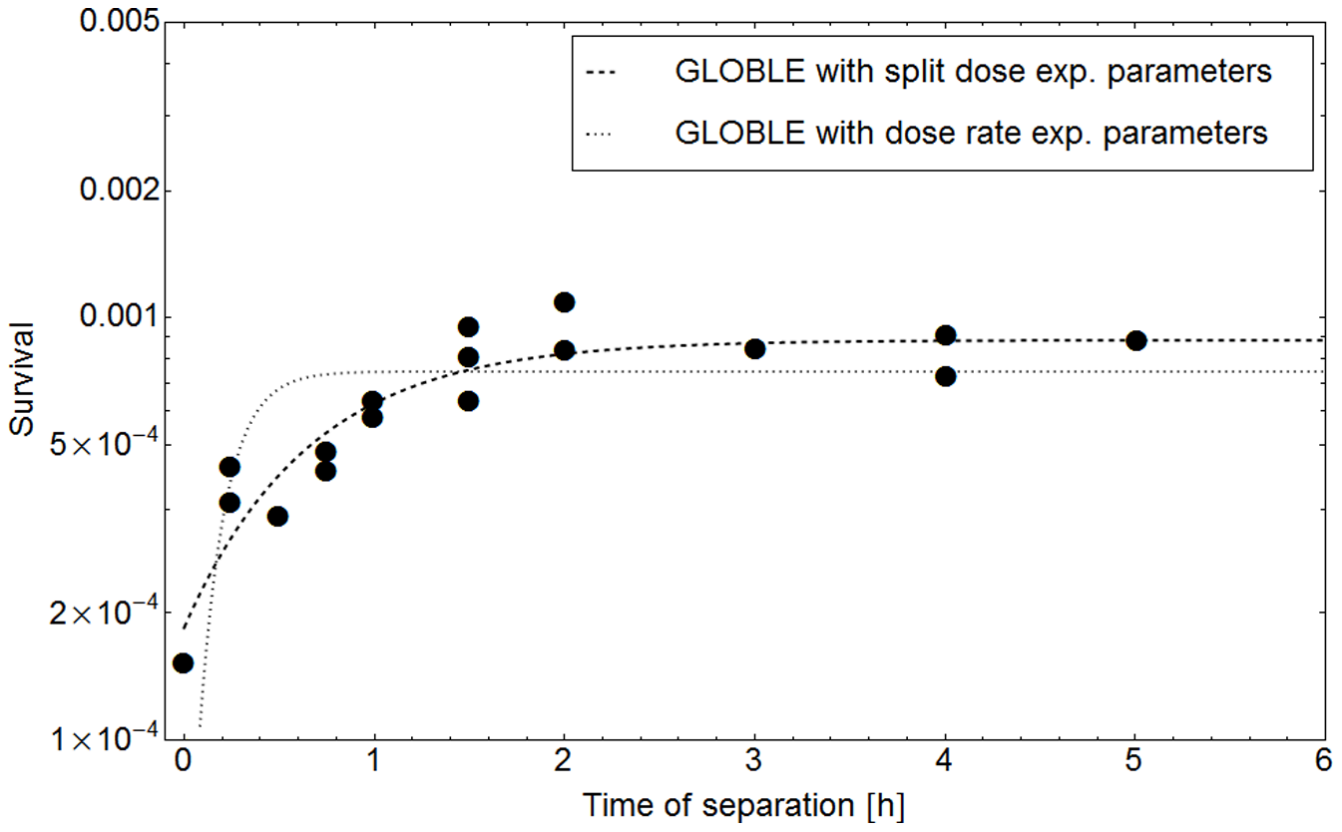
Prediction of split dose experiments with GLOBLE parameters derived from dose rate experiments. This figure shows the survival probability of a LL cell line measured in a split dose experiment with 5+5 Gy (dots) [37]. The data can be accurately reproduced by a fit of the GLOBLE (dashed line). Although the trend is the same, there are systematic deviations if survival probabilities are predicted with the GLOBLE under application of parameters gained from a dose rate experiment with the same cell line (dotted line).

The impact of the diverging half-life times computed on the basis of either dose rate or split dose experiments is visualized in figure 6. Here, the measured survival probabilities of an LL cell line after split dose experiments (dots) are compared to the survival probabilities one would predict with the GLOBLE under usage of the parameters gained in a dose rate experiment (dotted line). Clearly visible, the predicted survival probability reaches its maximum value at smaller times of separation between the two doses (∼0.5 h) than it is observed in the experiment (∼2 h). This is due to the fact that the maximum survival probability is achieved when a cell has enough time to recover from the first dose before the second one is given. If DSBs are repaired faster (smaller *HLT_i_*), the recovery takes less time and the maximum survival probability is reached at smaller times between the fractions, consequently.

To summarize, the differing values of *HLT_i_* found in dose rate and split dose experiments do not falsify the hypothesis that the characteristics of one cell line can be described with one set of parameters in the GLOBLE. The experimental uncertainties have to be taken into account here. Furthermore, the values of ε_i_ found with fits to the different experimental approaches are in the same order of magnitude (table 2); this even further supports the concept of the model.

### Analytical investigation

The analytical examination of the kinetically extended GLOBLE which has been presented in the results reveals that the model provides consistent formulations in all limits of applied doses and constant dose rates. It is demonstrated that in the limit of high dose rates the kinetic extension converges to the static version which was published and discussed in [17]. Therefore, the dynamics that are introduced for the kinetic extension complement the parent version in the right fashion.

### Agreement with empirical findings

As an empirical formulation of dose rate effects in cell survival curves, the Linear-Quadratic model was considered. The detailed analytical comparison of the GLOBLE with the Linear-Quadratic model for the case of a single photon dose treatment with constant dose rate has shown that the two models are approximately equivalent up to second order in dose and that deviations occur due to higher orders in the dose. The question which of the two models performs better in the description of experimental data must be answered elsewhere, potentially in the context of a more general model comparison involving the IR model and the LPL model.

For the description of dose rate effects observed in the incidence of deterministic effects after radiation exposures an empirically found linear relationship between a reference dose and the inverse of the dose rate was taken (equation (42)). This approach suggests that infinitely large doses are required for the incidence of a disease at very low dose rates and consequently it is clearly not suitable for the prediction of incidence probabilities of deterministic effects at dose rates & 3 Gy/h. It has been shown that the GLOBLE is in accordance with the empirical approach down to≈3 Gy/h and that the two formulations deviate at lower dose rates. Therefore, the GLOBLE provides an excellent mean to assess the considered clinical risks of exposures to radiation.

## Supporting Information

File S1 **Approximate closed form solution for the kinetically extended GLOBLE.**
(DOC)

## References

[1] Pop LA, Millar WT, van der Plas M, van der Kogel AJ (2000) Radiation tolerance of rat spinal cord to pulsed dose rate (PDR-) brachytherapy: the impact of differences in temporal dose distribution. Radiother Oncol 55(3): 301–15. 10869745 10.1016/s0167-8140(00)00205-x

[2] Stüben G, van der Kogel AJ, van der Schueren E (1997) Biological equivalance of low dose rate to multifractionated high dose rate irradiations: investigations in mouse lip mucosa. Radiother Oncol 42(2): 189–96. 9106929 10.1016/s0167-8140(96)01869-5

[3] Orton CG (2001) High-dose-rate brachytherapy may be radiobiologically superior to low-dose rate due to slow repair of late-responding normal tissue cells. Int J Radiat Oncol Biol Phys 49(1): 183–9. 11163513 10.1016/s0360-3016(00)00810-5

[4] Supe SS, Ganesh KM, Naveen T, Jacob S, Sankar BN (2006) Spinal cord response to altered fractionation and re-irradiation: radiobiological considerations and role of bioeffect models. J Cancer Res Ther 2(3): 105–18. 17998688 10.4103/0973-1482.27597

[5] Ling CC, Gerweck LE, Zaider M, Yorke E (2010) Dose-rate effects in external beam radiotherapy redux. Radiother Oncol 95(3): 261–8. 20363041 10.1016/j.radonc.2010.03.014

[6] Van Os R, Thames HD, Konings AWT, Down JD (1993) Radiation dose-fractionation and dose-rate relationships for long-term repopulating hemopoietic stem cells in a murine bone marrow transplant model. Radiat Res 136: 118–25. 8210327

[7] Van Dyk J, Mah K, Keane TJ (1989) Radiation-induced lung damage: dose-time-fractionation considerations. Radiotherapy and Oncology 14: 55–69. 2928557 10.1016/0167-8140(89)90009-1

[8] Thames HD, Withers HR, Peters LJ, Fletcher GH (1982) Changes in early and late radiation responses with altered dose fractionation: implications for dose-survival relationships. Int J Radiat Oncol Biol Phys 8: 219–26. 7085377 10.1016/0360-3016(82)90517-x

[9] Eguchi-Kasai K, Kosaka T, Sato K, Kaneko I (1991) Reparability of DNA double-strand breaks and radiation sensitivity in five mammalian cell lines. Int J Radiat Biol 59(1): 97–104. 1671079 10.1080/09553009114550091

[10] Menegakis A, Yaromina A, Eicheler W, Dörfler A, Beuthien-Baumann B, et al (2009) Prediction of clonogenic cell survival curves based on the number of residual DNA double strand breaks measured by *γ*H2AX staining. Int J Radiat Biol 85(11): 1032–41. 19895280 10.3109/09553000903242149

[11] Banáth JP, MacPhail SH, Olive PL (2004) Radiation sensitivity, H2AX phosphorylation, and kinetics of repair of DNA strand breaks in irradiated cervical cancer cell lines. Cancer Research 64: , 7144–9. 10.1158/0008-5472.CAN-04-1433 15466212

[12] Taneja N, Davis M, Choy JS, Beckett MA, Singh R, et al (2004) Histone H2AX phosphorylation as a predictor of radiosensitivity and target for radiotherapy. J Biol Chem 279: 2273–80. 14561744 10.1074/jbc.M310030200

[13] MacPhail SH, Banáth JP, Yu TY, Chu EH, Lambur H, Olive PL (2003) Expression of phosphorylated histone H2AX in cultured cell lines following exposure to X-rays. Int J Radiat Biol 79(5): 351–8. 12943243 10.1080/0955300032000093128

[14] Johnston PJ, Bryant PE (1994) A component of DNA double-strand break repair is dependent on the spatial orientation of the lesions within the higher-order structures of chromatin. Int J Radiat Biol 66: 531–6. 7983441 10.1080/09553009414551571

[15] Yokota H, van den Engh G, Hearst JE, Sachs RK, Trask BJ (1995) Evidence for the organization of chromatin in megabase pair-sized loops arranged along a random walk path in the human G0/G1 interphase nucleus. The Journal of Cell Biology 130: 1239–49. 7559748 10.1083/jcb.130.6.1239 PMC2120584

[16] Rogakou EP, Boon C, Redon C, Bonner WM (1999) Megabase chromatin domains involved in DNA double-strand breaks in vivo. J Cell Biol 146(5): 905–15. 10477747 10.1083/jcb.146.5.905 PMC2169482

[17] Friedrich T, Durante M, Scholz M (2012) Modeling cell survival after photon irradiation based on double-strand break clustering in megabase pair chromatin loops. Radiat Res 178: 385–94. 22998227 10.1667/RR2964.1

[18] Dahm-Daphi J, Dikomey E (1996) Rejoining of DNA double-strand breaks in X-irradiated CHO cells studied by constant- and graded-field gel electrophoresis. Int J Radiat Biol 69(5): 615–21. 8648249 10.1080/095530096145625

[19] Núñez MI, Villalobos M, Olea N, Valenzuela MT, Pedraza V, et al (1995) Radiation-induced DNA double-strand break rejoining in human tumour cells. British Journal of Cancer 71: 311–6. 7841046 10.1038/bjc.1995.62 PMC2033588

[20] Fowler JF, Welsh JS, Howard SP (2004) Loss of biological effect in prolonged fraction delivery. Int J Radiat Oncol Biol Phys 59(1): 242–9. 15093921 10.1016/j.ijrobp.2004.01.004

[21] Steel GG, Deacon JM, Duchesne GM, Horwich A, Kelland LR, Peacock JH (1987) The dose-rate effect in human tumour cells. Radiotherapy and Oncology 9: 299–310. 3317524 10.1016/s0167-8140(87)80151-2

[22] Peacock JH, Cassoni AM, McMillan TJ, Steel GG (1988) Radiosensitive human tumour cell lines may not be recovery deficient. Int J Radiat Biol 54(6): 945–53. 2903891 10.1080/09553008814552341

[23] Lea DE (1946) Actions of radiations on living cells. 1^st^ ed. London: Cambridge University Press.

[24] Catcheside DG, Lea DE, Thoday DM (1946) The production of chromosome structural changes in tradescantia microspores in relation to dosage, intensity and temperature. J Genet 47: 137–49. 21010996 10.1007/BF02986783

[25] Ostashevsky J (1998) A polymer model for the structural organization of chromatin loops and minibands in interphase chromosomes. Molecular Biology of the Cell 9: 3031–40. 9802894 10.1091/mbc.9.11.3031 PMC25584

[26] Solovjeva L, Svetlova M, Stein G, Chagin V, Rozanov Y, et al (1998) Conformation of replicated segments of chromosome fibres in human S-phase nucleus. Chromosome Research 6: 595–602. 10099872 10.1023/a:1009293108736

[27] Johnston PJ, Olive PL, Bryant PE (1997) Higher-order chromatin structure dependent repair of DNA double-strand breaks: modeling the elution of DNA from nucleoids. Radiat Res 148: 561–7. 9399701

[28] Johnston PJ, MacPhail SH, Banáth JP, Olive PL (1998) Higher-order chromatin structure dependent repair of DNA double-strand breaks: factors effecting elution of DNA from nucleoids. Radiat Res 149: 533–42. 9611091

[29] Elsässer T, Kraft-Weyrather W, Friedrich T, Durante M, Iancu G, et al (2010) Quantification of the relative biological effectiveness for ion beam radiotherapy: direct experimental comparison of proton and carbon ion beams and a novel approach for treatment planning. Int J Radiat Oncol Biol Phys 78(4): 1177–83. 20732758 10.1016/j.ijrobp.2010.05.014

[30] Friedrich T, Scholz U, Elsässer T, Durante M, Scholz M (2012) Calculation of the biological effects of ion beams based on the microscopic spatial damage distribution pattern. Int J Radiat Biol 88(1–2): 103–7. 21823820 10.3109/09553002.2011.611213

[31] Löbrich M, Rydberg B, Cooper PK (1995) Repair of x-ray-induced DNA double-strand breaks in specific Not I restriction fragments in human fibroblasts: joining of correct and incorrect ends. Proc Natl Acad Sci USA 92: 12050–4. 8618842 10.1073/pnas.92.26.12050 PMC40294

[32] Wells RL, Bedford JS (1983) Dose-rate effects in mammalian cells. Radiat Res 94: 105–34. 6856762

[33] Stackhouse MA, Bedford JS (1993) An ionizing radiation-sensitive mutant of CHO cells: irs-20. Radiat Res 136: 250–4. 8248482

[34] Nagasawa H, Chen DJC, Strniste GF (1989) Response of X-ray sensitive CHO mutant cells to γ radiation. Radiat Res 118: 559–67. 2727276

[35] Stisova V, Abele WH, Thompson KH, Bennett PV, Sutherland BM (2011) Response of primary human fibroblasts exposed to solar particle event protons. Radiat Res 176: 217–25. 21557667 10.1667/rr2490.1

[36] Kelland LR, Steel GG (1986) Dose-rate effects in the radiation response of four human tumour xenografts. Radiotherapy and Oncology 7: 259–68. 3809588 10.1016/s0167-8140(86)80037-8

[37] Stephens TC, Eady JJ, Peacock JH, Steel GG (1987) Split-dose and low dose-rate recovery in four experimental tumour systems. Int J Radiat Biol 52(1): 157–70. 10.1080/09553008714551581 3496307

[38] Yang X, Darling JL, McMillan TJ, Peacock JH, Steel GG (1990) Radiosensitivity, recovery and dose-rate effect in three human glioma cell lines. Radiotherapy and Oncology 19: 49–56. 2122495 10.1016/0167-8140(90)90165-s

[39] Ruiz de Almodóvar JM, Bush C, Peacock JH, Steel GG, Whitaker SJ, McMillan TJ (1994) Dose-rate effect for DNA damage induced by ionizing radiation in human tumor cells. Radiat Res 138: S93–6. 8146338

[40] Holmes A, McMillan TJ, Peacock JH, Steel GG (1990) The radiation dose-rate effect in two human neuroblastoma cell lines. Br J Cancer 62: 791–5. 2245172 10.1038/bjc.1990.381 PMC1971544

[41] Little MP (2003) Risks associated with ionizing radiation. British Medical Bulletin 68: 259–75. 14757722 10.1093/bmb/ldg031

[42] Fry RJM (2001) Deterministic effects. Health Physics 80(4): 338–43. 11281201 10.1097/00004032-200104000-00009

[43] Edwards AA, Lloyd DC (1998) Risks from ionising radiation: deterministic effects. J Radiol Prot 18(3): 175–83. 9791808 10.1088/0952-4746/18/3/004

[44] Bentzen SM, Skoczylas JZ Bernier J (2000) Quantitative clinical radiobiology of early and late lung reactions. Int J Radiat Biol 76(4): 453–62. 10815624 10.1080/095530000138448

[45] Oliver R (1964) A comparison of the effects of acute and protracted gamma-radiation on the growth of seedlings of Vicia Faba. Part II. Theoretical calculations. Int J Radiat Biol 8(5): 475–88. 10.1080/09553006414550581 14248556

[46] Curtis SB (1986) Lethal and potentially lethal lesions induced by radiation - a unified repair model. Radiat Res 106: 252–70. 3704115

[47] Johnston PJ, MacPhail SH, Stamato TD, Kirchgessner CU, Olive PL (1998) Higher-order chromatin structure-dependent repair of DNA double-strand breaks: involvement of the V(D)J recombination double-strand break repair pathway. Radiat Res 149: 455–62. 9588356

[48] Gauter B, Zlobinskaya O, Weber KJ (2002) Rejoining of radiation-induced DNA double-strand breaks: pulsed-field electrophoresis analysis of fragment size distributions after incubation for repair. Radiat Res 157: 721–33. 12005552 10.1667/0033-7587(2002)157[0721:roridd]2.0.co;2

[49] Tommasino F, Friedrich T, Scholz U, Taucher-Scholz G, Durante M, Scholz M (2013) A DNA double-strand break kinetic rejoining model based on the Local Effect Model. Radiat Res 180: 524–38. 24138482 10.1667/RR13389.1

[50] Nikjoo H, O'Neill P, Wilson WE, Goodhead DT (2001) Computational approach for determining the spectrum of DNA damage induced by ionizing radiation. Radiat Res 156: 577–83. 11604075 10.1667/0033-7587(2001)156[0577:cafdts]2.0.co;2

[51] Ottolenghi A, Merzagora M, Tallone L, Durante M, Paretzke HG, Wilson WE (1995) The quality of DNA double-strand breaks: a Monte Carlo simulation of the end-structure of strand breaks produced by protons and alpha particles. Radiat Environ Biophys 34: 239–44. 8749062 10.1007/BF01209749

[52] Ward JF (1994) The complexity of DNA damage: relevance to biological consequences. Int J Radiat Biol 66(5): 427–32. 7983426 10.1080/09553009414551401

[53] Friedland W, Jacob P, Paretzke HG, Ottolenghi A, Ballarini F, Liotta M (2006) Simulation of light ion induced DNA damage patterns. Radiat Prot Dosimetry 122: 116–20. 17166872 10.1093/rpd/ncl451

[54] Löbrich M, Shibata A, Beucher A, Fisher A, Ensminger M, et al (2010) γH2AX foci analysis for monitoring DNA double-strand break repair – strengths, limitations and optimization. Cell Cycle 9(4): 662–9. 20139725 10.4161/cc.9.4.10764

[55] Neumaier T, Swenson J, Pham C, Polyzos A, Lo AT, et al (2012) Evidence for formation of DNA repair centers and dose-response nonlinearity in human cells. PNAS 109(2): 443–8. 22184222 10.1073/pnas.1117849108 PMC3258602

