# Supplementary material for: A Model of Photon Cell Killing Based on the Spatio-Temporal Clustering of DNA Damage in Higher Order Chromatin Structures
Source: PLoS One. 2014 Jan 2;9(1):e83923. doi: 10.1371/journal.pone.0083923 (PMC3879277; doi:10.1371/journal.pone.0083923)
Supplement: File S1 — Approximate closed form solution for the kinetically extended GLOBLE. (DOC) [file pone.0083923.s001.doc]

**Supplement 1**

**File S1. Approximate closed form solution for the kinetically extended GLOBLE**

The set of coupled differential equations (13) – (17) has no closed form solution and thus numerical approaches have to be applied. However, detailed consideration of the specific features of clustered DSBs motivates the introduction of an approximation which simplifies the set of equations and leads to a closed form solution.

First, the induction of further DSBs within a DNA loop already containing a clustered DSB does not lead to a higher lethality according to the definition of clustered DSBs made in this publication. Thus, the relevant kinetic aspect is the repair of a clustered DSB leading to either a lethal event or a viable outcome. Since correct repair or viable misrepair is assumed to directly lead to the “ground state” *f_0_*, the corresponding domain re-enters the circuit again and is in principle susceptible to induction of additional DSBs. However, for the relevant dose levels considered here, the probability to induce additional isolated DSBs in domains that have recovered from a former clustered DSB is very unlikely since the fraction of clustered DSBs is comparably low and the fraction of recovered domains even lower due to a relatively large value of ε_c_. In combination with the low lethality of isolated DSBs, the impact of domains recovered from clustered DSBs is expected to be hardly visible. Finally, although clustered DSBs show a higher lethality and thus in principle might have a higher impact, the probability to have a second clustered DSB in a loop recovered from a first clustered DSB is even much lower than that of an additional isolated DSB in the relevant dose range, and therefore this case can be also neglected.

Referring to these findings, clustered DSBs should have little influence on the dynamics involved in the processes running during irradiation and survival probabilities should hardly depend on the corresponding half-life time *HLT_c_*, consequently. To test the insignificance of *HLT_c_* in its whole range of possible values, survival probabilities predicted in the limiting cases of *HLT_c_* = *HLT_i_* (lower bound) or *HLT_c_* $\to\infty$ (upper bound) were compared to survival probabilities predicted with *HLT_c_* = 5 h, a value which is reasoned by experiments. In the range of doses, dose rates and parameter values prevailing in photon experiments (*D* < 100 Gy, < 100 Gy/h, 0.0001 < ε_i_ < 0.2, 0.05 < ε_c_ < 0.4, 0.05 h < *HLT_i_* < 5 h) the relative deviation is less than 4%.

As a consequence from the low impact of *HLT_c_* on cell survival probabilities, all terms emanating from the dynamics of clustered DSBs in (13) – (17) might be neglected in a first approximation, which then leads to a new set of only four differential equations:

(43)

(44)

(45)

$\frac{dl_{i}}{dt}=m_{i}f_{i}$. (46)

This set allows for a closed form solution for the levels *f_0_, f_i_* and *f_c_* :

$f_{0}(t)=\frac{1}{2}e^{-\frac{t}{2}y}(1+e^{-tx}+\frac{r}{x}(1-e^{-tx}))$ (47)

(48)

(49)

with

$r=m_{i}+r_{i}$; ; . (50)

From these equations closed form solutions for *l_i_, l_c_* and the survival can be derived.

To conclude, one may simply use the closed form solution of the GLOBLE to get a very good approximation for the cell survival without big computation efforts. Furthermore, since the impact of *HLT_c_* on cell survival is negligible in the whole range of possible values, one may set this parameter to a fixed value (experiments suggest 5 h) during fits to measured data without causing distortions.
